# RaceRunning training improves stamina and promotes skeletal muscle hypertrophy in young individuals with cerebral palsy

**DOI:** 10.1186/s12891-020-03202-8

**Published:** 2020-03-27

**Authors:** Emma Hjalmarsson, Rodrigo Fernandez-Gonzalo, Cecilia Lidbeck, Alexandra Palmcrantz, Angel Jia, Ola Kvist, Eva Pontén, Ferdinand von Walden

**Affiliations:** 1 Neuropediatrics Unit, Department of Women’s and Children’s Health, Karolinska Institutet, Tomtebodavägen 18a, 171 77 Stockholm, Sweden; 2 Allied Health Professionals Function, Medical Unit Occupational Therapy and Physiotherapy, Karolinska University Hospital, Stockholm, Sweden; 3 Department of Laboratory Medicine, Division of Clinical Physiology, Karolinska Institutet, and Unit of Clinical Physiology, Karolinska University Hospital, Stockholm, Sweden; 4 Department of Pediatric Orthopaedic Surgery, Karolinska University Hospital, Stockholm, Sweden; 5 Department of Pediatric Radiology, Karolinska University Hospital, Stockholm, Sweden; 6 Clinical Pediatrics Unit, Department of Women’s and Children’s Health, Karolinska Institutet, Stockholm, Sweden

**Keywords:** Aerobic exercise, Cardiorespiratory endurance, Cerebral palsy, Skeletal muscle, Skeletal muscle hypertrophy

## Abstract

**Background:**

Individuals with cerebral palsy (CP) are less physically active, spend more time sedentary and have lower cardiorespiratory endurance as compared to typically developed individuals. RaceRunning enables high-intensity exercise in individuals with CP with limited or no walking ability, using a three-wheeled running bike with a saddle and a chest plate for support, but no pedals. Training adaptations using this type of exercise are unknown.

**Methods:**

Fifteen adolescents/young adults (mean age 16, range 9–29, 7 females/8 males) with CP completed 12 weeks, two sessions/week, of RaceRunning training. Measurements of cardiorespiratory endurance (6-min RaceRunning test (6-MRT), average and maximum heart rate, rate of perceived exertion using the Borg scale (Borg-RPE)), skeletal muscle thickness (ultrasound) of the thigh (vastus lateralis and intermedius muscles) and lower leg (medial gastrocnemius muscle) and passive range of motion (pROM) of hip, knee and ankle were collected before and after the training period.

**Results:**

Cardiorespiratory endurance increased on average 34% (6-MRT distance; pre 576 ± 320 m vs. post 723 ± 368 m, *p* < 0.001). Average and maximum heart rate and Borg-RPE during the 6-MRT did not differ pre vs. post training. Thickness of the medial gastrocnemius muscle increased 9% in response to training (*p* < 0.05) on the more-affected side. Passive hip flexion increased (*p* < 0.05) on the less-affected side and ankle dorsiflexion decreased (*p* < 0.05) on the more affected side after 12 weeks of RaceRunning training.

**Conclusions:**

These results support the efficacy of RaceRunning as a powerful and effective training modality in individuals with CP, promoting both cardiorespiratory and peripheral adaptations.

## Background

Cerebral palsy (CP) is the most common childhood motor disability, with a prevalence of around 2.0–2.5 per 1000 live births [1]. The damage to the brain is permanent and non-progressive, but the severity and nature of the clinical manifestations often change over time [2]. With the Gross Motor Function Classification System (GMFCS) children and youth with CP can be classified on a 5-level scale based on their self-initiated movement with particular emphasis on sitting, walking, and wheeled mobility [3, 4]. While individuals functioning at GMFCS level I participate in a great variety of activities, children with more severe motor function difficulties (GMFCS IV-V) have restricted options for physical activity participation including hydrotherapy, horseback riding and boccia, all examples of activities with low cardiorespiratory demands. Individuals with CP are known to be less physically active and spend more time sedentary as compared to typically developing (TD) individuals [5]. The level of physical activity negatively correlates to motor function as classified according to the GMFCS classification system, both in terms of physical education participation at school and regular physical activity during leisure time [6].

Muscle mass is significantly lower in children with CP as compared to TD children, including muscles in the lower limbs [7, 8]. Moreover, individuals with CP typically have lower cardiorespiratory endurance as compared to TD individuals [9]. It is well known that aerobic capacity increases with endurance training in TD individuals and positively influences quality of life and overall health status [10]. CP is associated with an increased risk of multiple disorders linked to premature aging and inactivity such as coronary artery disease and type 2 diabetes [11, 12]. Stimulating individuals with CP to regularly participate in moderate to high-intensity activities would likely increase physical fitness and thereby reduce the risk of disease, especially since it has been shown that physical activity in adults with CP is related to their physical activity as adolescents [13]. Secondary musculoskeletal problems including decreased range of motion are common in children with CP [14, 15]. Reduced knee and hip joint extension are known to influence function and are associated with gait inefficiency in adolescents with CP [16]. Moreover, in a study by van der Linden and colleagues reduced pROM in knee extention and skeletal muscle spasticity were found to be negatively associated with RaceRunning performance in CP [17].

The RaceRunner is a three-wheeled running bike with a saddle and a chest plate for support but no pedals [17–19] and can be used by individuals in GMFCS level I-V (see supplementary figure 1). It enables high-intensity exercise for individuals with CP who (may) have limited or no walking ability [18]. However, training adaptations using this type of exercise are unknown.

Therefore, we performed a 12-week RaceRunning training study to address the following research questions: 1) Does cardiorespiratory endurance increase with training in individuals with CP? 2) Does skeletal muscle thickness increase with RaceRunning training? 3) Is the passive range of motion of the hip, knee and ankle affected by RaceRunning training?

## Methods

### Study design

**Fig. 1:**
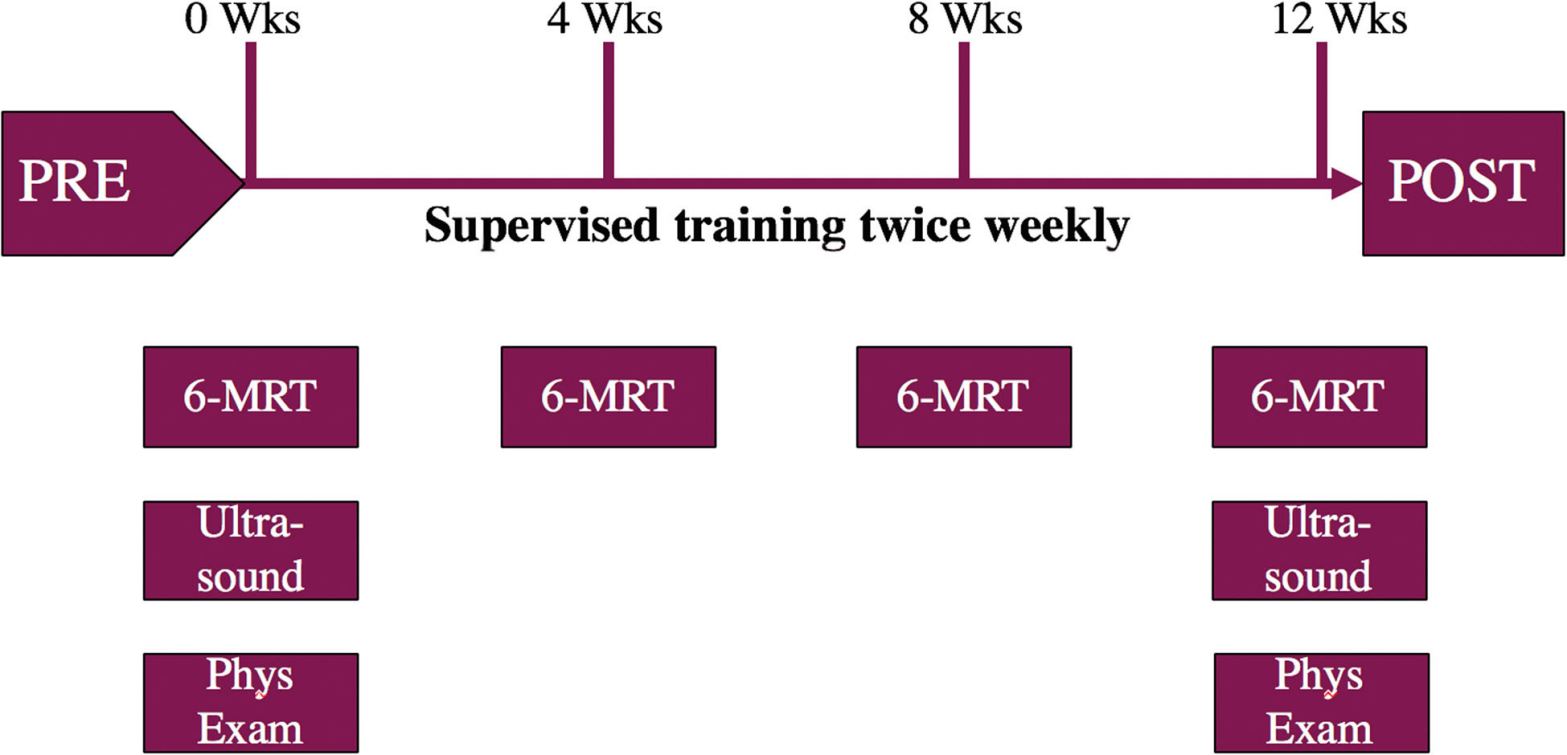
Schematic timeline of experimental setup. PRE = before training period, POST = after training period, Wks = Weeks, 6-MRT = 6-min RaceRunning test, Phys Exam = Physical examination

This intervention study aims to evaluate the effects of RaceRunning training twice per week for 12 weeks in children, adolescents and young adults with CP. The tests and evaluations took place during two weeks before and two weeks after the training period. In addition, 6-min RaceRunning test (6-MRT) [18] was performed at week four and eight (Fig. 1).

### Participants and recruitment

Fifteen (*n* = 15) children/adolescents/young adults (mean age 16, range 9–29, 7 females/8 males) with CP completed 12 weeks, two sessions per week, of RaceRunning training (subject characteristics in Table 1). Recruitment of participants took place via RaceRunning sport associations in three cities in Sweden; Uppsala, Stockholm and Västerås, and were based on interest and inclusion/exclusion criteria. Inclusion criteria were children and young adults with CP, GMFCS I-IV within the age span 7–35 years. In order to avoid initial learning effects, and/or effects of adjusting the RaceRunner to the individual, all participants were accustomed to the sport for at least three months before the intervention. The amount of previous practice varied between participants (three months to > three years), but none had followed any structured, supervised training program before the commencement of the study. Exclusion criteria were orthopedic surgery or injections of botulinum toxin during or three months prior to the study. In addition, interventions aiming at reducing skeletal muscle spasticity such as history of selective dorsal rhizotomy or intrathecal baclofen were also seen as exclusion criteria. Eighteen participants were enrolled to participate in the study, but three participants were excluded due to additional diagnoses or incomplete training and/or evaluations.

**Table 1:** Subject characteristics

Variable	Description of participants (*n* = 15)
Age	16 [9–29]
Gender (female / male)	7 / 8
BMI	18,6 [13,5 – 22,6]
Type of CP; Spastic / Dyskinetic / Ataxia	9 / 5 / 1
GMFCS-level; I / II / III / IV	1 / 3 / 4 / 7
FMS 5 m 1 / 2 / 3 / 4 / 5 / 6	8 / 0 / 2 / 0 / 4 / 1
FMS 50 m 1 / 2 / 3 / 4 / 5 / 6	8 / 4 / 0 / 0 / 3 / 0
FMS 500 m 1 / 2 / 3 / 4 / 5 / 6	12 / 1 / 0 / 0 / 2 / 0

Subject characteristics, mean [range]. *BMI* Body Mass Index, *GMFCS* Gross Motor Function Classification System, *FMS* Functional Mobility Scale, with number of participants in each category

### Intervention

The training period consisted of supervised RaceRunning exercise twice per week for 12 weeks, one h per session, together with the local club at an indoor track and field facility. Participants were instructed to follow a specific training program during each session. The program consisted of five blocks: warm-up (light jog), short distance intervals (sprints of 40-100 m), long distance intervals (continuous running for three to ten min at medium to high speed), technique exercises (60 m slalom course with eight to ten markers or 60 m run alternating using only the right/left leg), and wind-down (light jog). Participants trained in groups consisting of 5–15 individuals. Participants were encouraged to work at an intensity that made them sweat and out of breath, but no specific target heart rate or target level of perceived exertion (Borg-RPE) was set during the training sessions. Depending on the training site (Stockholm, Uppsala or Västerås) the coach was either a physiotherapist or bachelor/master degree in sports science. One training session per week was recorded using a trip computer with speed sensor (Garmin Edge 25, Garmin, United States) and a chest strap (Garmin) enabling data collection of distance travelled (meters), time spent in motion with the RaceRunner (minutes) during each session, speed (meters/second), and average and maximum heart rate during the training session (beats/minute) [20, 21].

### 6-min RaceRunning test for cardiorespiratory endurance

To assess gains in cardiorespiratory endurance the 6-min RaceRunning test (6-MRT) was used [18]. This test measures the total distance covered with the RaceRunner during six min. The participants were allowed to warm up for five min on the RaceRunner prior to the test and were then instructed to run as far as possible during six min. Participants performed the 6-MRT a total of four times; 1) before the training period, 2) at week four, 3) week eight and 4) after the training period. Distance, and average and maximal heart rate were measured at all time points. Data were collected as described above using the trip computer (Garmin Edge 25) and the distance was verified using a measuring wheel. Level of perceived exertion (Borg-RPE) was rated using the 6–20 Borg scale [22]. Two participants did not perform the 12w 6-MRT. Data from the eight week 6-MRT were used for pre vs post training evaluation for these participants.

### Ultrasound examination for skeletal muscle thickness

Muscle thickness was assessed in the calf (medial gastrocnemius muscle) and thigh (vastus lateralis and intermedius muscles of the quadriceps) in both legs using ultrasound (Siemens acuson s2000, Siemens, Erlangen, Germany) with a built-in function for measurements of skeletal muscle. Several images (5–8) were taken of each muscle, to ensure consistency of measured values. The ultrasound measurements were performed before and after 12 weeks of RaceRunning training by the same radiologist. The leg with the thinnest medial gastrocnemius muscle was denoted as the more affected leg [23].

### Physical examination

Participants underwent a physical examination of the lower limbs by the same physiotherapist before and after the training period. At the first examination, data collection included classification of motor function using the GMFCS [4]. Passive range of motion (pROM) was measured in the hip, knee and ankle with a goniometer in standardized positions [24]. In addition, muscle spasticity was assessed according to the Modified Ashworth scale [25] with the grading 0, 1, + 1, 2, 3 or 4. After 12 weeks of RaceRunning training, the physical examination was performed in 14 participants. One participant did not come to the post physical examination and was therefore excluded from the pROM analysis.

### Statistics

Data corresponding to 6-MRT, heart rate (average and maximum) and distance covered were analyzed using a general linear model with repeated measures, with time (pre, 4-wk, 8-wk, 12-wk) as within-subject factor. Potential differences between pre- and post-training in muscle thickness were assessed employing paired t-tests. Top speed was analyzed with a general linear model with repeated measures, with time (pre, 12-wk) as within-subject factor. Normal distribution of data was verified using the Shapiro-Wilk test. A Wilcoxon signed Rank test was used to compare pROM and a Fischer’s exact test was used to compare spasticity in the lower limbs before and after the training period. Data were analyzed using SPSS v.25 (Chicago, Illinois) and are presented as mean ± SD unless stated otherwise. Statistical significance was set at *p* < 0.05.

## Results

### Cardiorespiratory endurance (or RaceRunning capacity)

Participants were on average in motion 25 min (range 4–42 min) during the training sessions. The cardiorespiratory training intensity averaged 136 heart beats per minute (bpm) (range 112–166 bpm), i.e. 69% (range 58-82%) of estimated maximum heart rate (208–0.7xage; Table 2). Average maximum heart rate during the training sessions was 168 bpm (range 139–197 bpm) or 82% (range 74–98%) of estimated maximum heart rate.

**Table 2:** Training intensity

Subject	Age	Type of CP	GMFCS	Average time in motion per training hour (minutes)	Average HR (bpm) (% of age corrected HRmax)	Average of HRmax (bpm) (% of age corrected HRmax)	Top measured HR (bpm) (% of age corrected HRmax)
1	10–14	Dyskinetic	4	23	136 (69%)	169 (85%)	208 (105%)
2	10–14	Dyskinetic	4	29	123 (61%)	164 (82%)	183 (91%)
3	15–19	Dyskinetic	4	22	127 (65%)	158 (81%)	189 (97%)
4	15–19	Dyskinetic	4	34	135 (69%)	170 (87%)	213 (109%)
5	20–24	Dyskinetic	3	42	149 (77%)	175 (90%)	189 (97%)
6	25–29	Spastic bi	2	24	154 (81%)	176 (93%)	185 (97%)
7	25–29	Spastic bi	4	4	115 (61%)	139 (74%)	163 (87%)
8	20–24	Spastic bi	3	29	112 (58%)	156 (81%)	198 (103%)
9	5–9	Spastic uni	1	21	166 (82%)	186 (92%)	204 (101%)
10	10–14	Spastic bi	3	24	140 (70%)	166 (83%)	184 (92%)
11	10–14	Spastic bi	2	31	158 (79%)	197 (98%)	208 (104%)
12	10–14	Spastic bi	3	28	148 (74%)	176 (88%)	196 (99%)
13	10–14	Spastic bi	4	17	132 (66%)	170 (85%)	196 (98%)
14	10–14	Spastic bi	4	24	122 (61%)	152 (76%)	188 (94%)
15	5–9	Ataxic	2	18	126 (62%)	161 (80%)	196 (97%)
Mean value				25	136 (69%)	168 (85%)	193 (98%)
Range: min – max				4–42	112–166 (58–82%)	139–197 (74–98%)	163-213 (87–109%)

*GMFCS* Gross Motor Function Classification System, *HR* heart rate, *bpm* beats per minute, *HRmax* maximal heart rate. Age stated in 5-year intervals to preserve participant integrity and anonymity

**Fig. 2:**
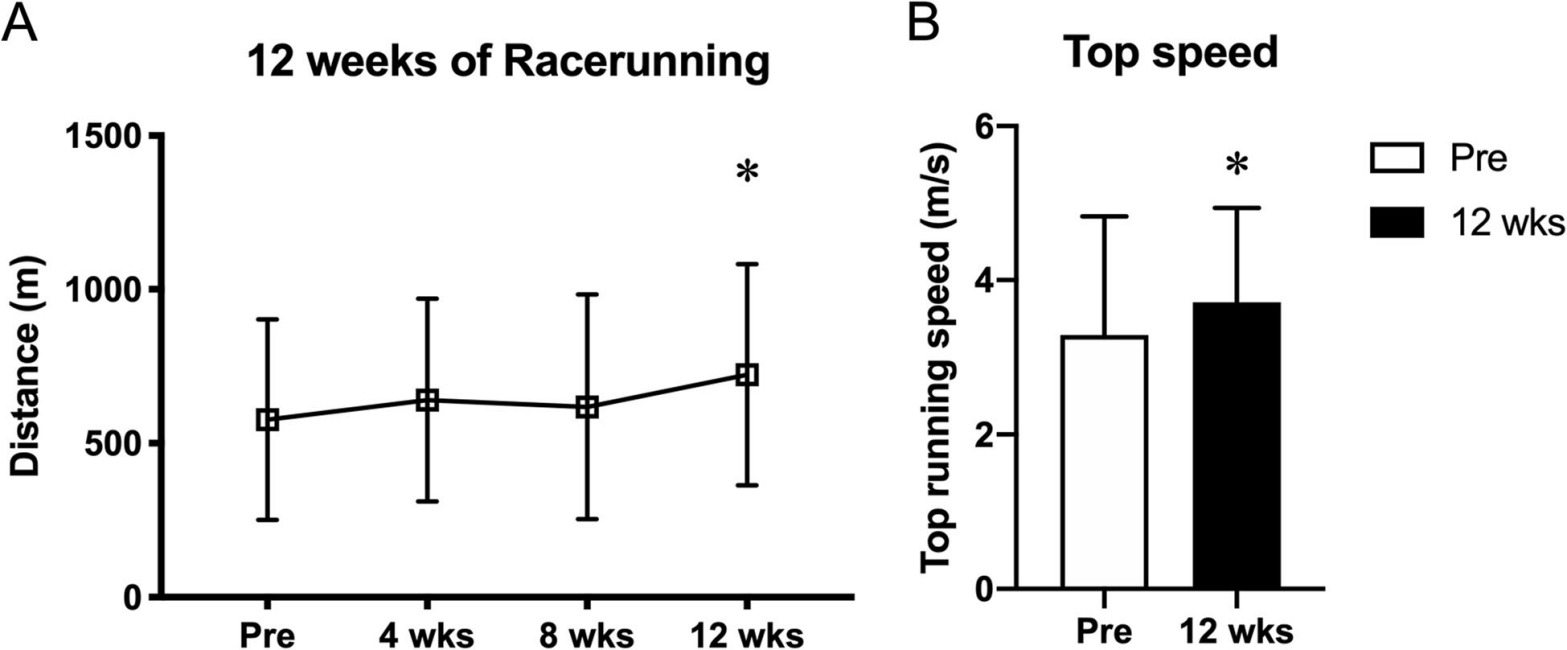
**a** Distance on the 6-min RaceRunning test during the training period (0,4,8 and 12 weeks). * denotes significantly different from Pre (*p* < 0.001). **b** Top running speed pre and after 12 weeks of RaceRunning training. * denotes significantly different from Pre (*p* < 0.05)

Following 12-weeks of RaceRunning training, all participants had increased their running distance by on average 34% (6-min RaceRunning test distance; pre 576 ± 320 m vs. post 723 ± 368 m, *p* < 0.001, Fig. 2a). Mean top speed also increased pre vs. post 12-weeks of RaceRunning training (3.3 ± 1.5 m/s vs. 3.7 ± 1.2 m/s, *p* < 0.05, Fig. 2b) corresponding to an average 21% (range − 6 to + 85%). Average (149 ± 35 vs. 156 ± 27, *p* = 0.667) and maximum (171 ± 27 vs. 177 ± 19, *p* = 0.840) heart rate during the 6-MRT did not differ pre vs. post training. Similarly, the level of perceived exertion during the 6-MRT, as rated using the Borg scale, did not change (pre-6MRT-score 8.2 ± 3.1and 8.1 ± 3.5, *p* = 0.491 and after-6MRT-score 14.7 ± 3.8 and 15.1 ± 3.7, *p* = 0.947).

### Skeletal muscle thickness

**Fig. 3:**
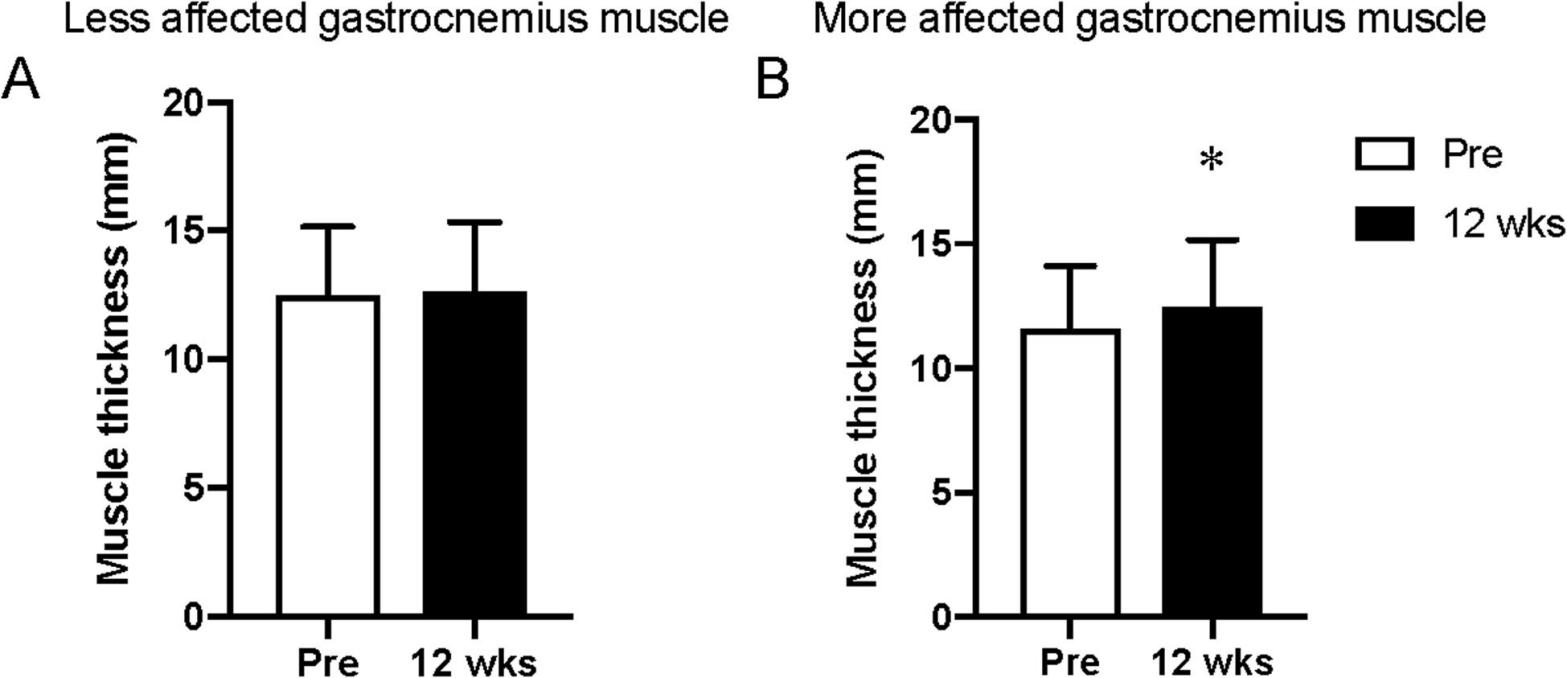
**a**: Muscle thickness of the **a**) less affected and **b**) more affected medial gastrocnemius muscle pre and after 12 weeks of Racerunning training. * denotes significantly different from Pre (*p* < 0.05)

Thickness of the medial gastrocnemius muscle in the more affected leg increased significantly after 12 weeks of RaceRunning training (11.6 ± 2.7 mm vs. 12.4 ± 2.6 mm, *p* = 0.005, Fig. 3b), but no significant change was found in the same muscle of the less affected leg (12.5 ± 2.7 mm vs. 12.6 ± 2.6 mm, *p* = 0.251, Fig. 3a). No significant change was found in thickness of the vastus lateralis and intermedius muscles in any leg (more-affected 23.6 ± 6.1 mm vs. 23.6 ± 7.0 mm, *p* = 0.389; less affected 26.6 ± 6.5 mm vs. 26.0 ± 7.0 mm, *p* = 0.269).

### Physical examination: passive range of motion and spasticity

After the training period, passive hip flexion in the less-affected leg increased as compared to pre-training measurements (median [min, max] 125° [70°, 150°] vs. 130° [100°, 150°], *p* = 0.015), but no significant difference was found in the more-affected hip (128° [80°, 150°] vs. 125° [110°, 150°], *p* = 0.080). Passive ankle dorsiflexion in the more-affected limb decreased pre vs. post training (10° [− 15°, 35°] vs 10° [− 20°, 35°], *p* = 0.026). No significant difference was found in the less-affected ankle (10° [− 5°, 30°] vs. 10° [0°, 35°], *p* = 0.218, Table 3). There was no significant difference in spasticity in the lower limbs before and after the training period (Supplementary table 1).

**Table 3:** passive range of motion pre vs post intervention

	More-affected leg			Less-affected leg		
	Before (*n* = 14)	After (*n* = 14)	*p-value*	Before (*n* = 14)	After (*n* = 14)	*p-value*
pROM, median [min, max]						
Hip flexion	128 [80, 150]	125 [110, 150]	0.080	125 [70, 150]	130 [100, 150]	**0.015**
Hip extension	0 [−30, 15]	2 [−20, 10]	0.200	0 [−30, 20]	0 [−20, 15]	0.643
Hamstrings lenght	125 [100, 145]	122 [100, 150]	0.560	125 [105, 160]	125 [110, 160]	0.660
Knee flexion	155 [60, 155]	155 [85, 155]	0.414	155 [80, 155]	155 [75, 155]	0.655
Knee extension	−15 [−45, 5]	−18 [−40, 5]	0.107	−10 [−40, 10]	−15 [−45, 10]	0.248
Ankle dorsiflexion	10 [−15, 35]	10 [−20, 35]	**0.026**	10 [−5, 30]	10 [0, 35]	0.218

Passive range of motion (pROM) in median [min, max] degrees, before and after a training period with RaceRunning (*p*-level < 0.05 indicated in bold). Negative values (−) indicate range of motion to less than neutral position of the joint

## Discussion

Individuals with CP are known to be less physically active, spend more time sedentary and have lower cardiorespiratory endurance as compared to TD individuals. In order to counteract diseases associated with poor muscle health and a sedentary lifestyle, e.g. type-2 diabetes and cardiovascular disease, physical activity modalities allowing for varying degrees of motor disability are warranted. There are currently not many physical exercise alternatives that promote cardiovascular adaptations for people with severe disabilities. RaceRunning enables high-intensity exercise in individuals with CP with limited or no walking ability. Our data support the use of RaceRunning to increase cardiorespiratory fitness and promote skeletal muscle hypertrophy in affected limbs in individuals with CP.

The main finding of the present study was that 12 weeks of RaceRunning training twice per week improves cardiorespiratory endurance on average 34% as compared to pre-training values. All participants included in the intervention improved their performance on the 6-MRT, whereas average and maximal heart rate, as well as pre-post ratings on the Borg-RPE scale remained unchanged. We interpret this as evidence in favor of cardiorespiratory adaptation to exercise, as longer distance traveled during the 6-MRT at a fixed heart rate suggest either central adaptation (stroke volume) and/or peripheral adaptation (hemoglobin, myoglobin, mitochondrial content, muscle hypertrophy). Two studies investigating the results of aerobic training in children with CP have also reported that while oxygen consumption, VO_2max_, increased, the heart rate during the test situation remained the same [26, 27]. Our Borg-RPE data further support this view, as the Borg-RPE remained constant before and after the 12-week training period, whereas the performance on the 6-MRT improved. Our results are well in line with previous training studies investigating the effects of endurance exercise interventions in individuals with CP (GMFCS I-II). Training duration of six weeks to three months has been reported to result in improvements in peak oxygen consumption of 18–23% in adolescents with CP [27–29]. Longer periods of training (up to nine months) display 35–41% increments in aerobic performance [30, 31], suggesting a positive relationship between training duration and improvements in cardiorespiratory endurance. Berg performed one of the first longer training studies in school children with CP (*n* = 22), investigating the result of up to 16 months (range 1.5 months to 16 months) of aerobic training [32]. Twenty out of 22 participants increased their VO_2max_; 12 participants improved > 25% and the remaining eight participants by 10–15%. A linear relationship between training duration and increase in VO_2_ max as a result of training (correlation coefficient 0.68) was observed in the study by Berg. With respect to recommendations by the American College of Sports Medicine (ACSM) regarding training frequency and duration [33], our study with a training intensity of 65% of age-corrected maximum heart rate, should have been performed three times per week rather than the actual two times per week. Training at this frequency is often hard for schoolchildren with CP who depend heavily on their families. However, as pointed out by Verschuren and colleagues, previous studies in which the actual training frequency did not meet the minimal recommendations for people with CP, have nevertheless shown remarkable improvements in aerobic capacity [9]. This suggests that for sedentary and deconditioned individuals with CP, an initial training dose of one to two times per week is likely sufficient, a frequency that can gradually be increased as adaptations occur. Our data supports this point of view and provide evidence that, given sufficient work intensity, a frequency of two times per week is effective in promoting cardiorespiratory endurance adaptations.

In addition to improving cardiorespiratory fitness, 12 weeks of RaceRunning training resulted in 9% hypertrophy of the medial gastrocnemius muscle on the more affected side. This was an intriguing finding, as endurance exercise such as running is not traditionally seen as an activity promoting skeletal muscle growth. However, recent evidence suggests that low load exercise such as walking and biking can have anabolic properties in older, untrained individuals [34]. Harber and co-workers investigated the effect of a 12 week, twice per week, cycle ergometer training protocol at 60–80% VO_2_-workload on skeletal muscle size on > 70-year-old women [35]. Skeletal muscle fiber size increased on average 12% as a result of training in this cohort. Similarly, a study investigating the effects of six months of walking training on muscles in the lower limb reported increases in skeletal muscle thickness in sedentary to moderately active older adults [36]. On the contrary, active older men and women did not increase cross-sectional area (CSA) of the vastus lateralis muscle after ten weeks of walking training [37]. Collectively, these studies suggest that ambulatory activity with sufficient intensity (> 60% of the heart rate reserve) can result in increments of skeletal muscle size in older adults, but that the outcome is influenced by initial physical activity levels. It is well established that individuals with CP are known to be less physically active and spend more time sedentary as compared to typically developed individuals [5]. Moreover, skeletal muscle in CP was recently referred to as a model of premature aging in relation to sarcopenia and skeletal muscle dysfunction [38]. In this narrative review article, the authors highlighted evidence of poor muscle quality in individuals with CP, e.g. increased intramuscular collagen content and inter- and intramuscular fat, and early atrophy predisposing for a sedentary life in adulthood. This is exemplified by that the vast majority (75%) of ambulatory individuals with CP eventually stop walking by choice at adult age because of fatigue, inefficient ambulation and/or because using a wheelchair expanded their access to community activities [39]. Thus, we propose that a combination of a low level of every day activity and a low starting point with respect to skeletal muscle thickness might explain the hypertrophic response of the calf muscle on the more affected side following 12 weeks of RaceRunning training.

Van der linden and colleagues have reported that both reduced joint range of motion and increased muscle spasticity in the lower limb were negatively associated with speed [17]. In our study, we show that all participants, irrespectively of contractures and/or spasticity around the knee and/or ankle joint, where able to increase 6–MRT distance and top RaceRunning speed. Passive hip flexion increased modestly after the training period. On the contrary, dorsiflexion of the ankle joint decreased after training, indicating that we cannot expect to slow down contracture formation simply by stimulating individuals with CP to participate in endurance sports. It should be noted that all changes in pROM pre vs. post training were small and that the clinical significance of these changes in degrees of movement, both positive and negative around the joint, can be questioned. Larger studies that monitor individuals over a longer period of time are needed to explore contracture formation and the effect of exercise induced skeletal muscle remodeling in cerebral palsy.

The current report should be viewed upon in the light of the following limitations. Our number of participants were small and heterogeneous with respect to RaceRunning experience and severity of the motor impairment. Therefore, future studies should aim at enrolling a larger number of individuals, with a more homogenous training background and include a control group. Likewise, as this was a real-world set study and not a hospital run intervention, all our participants were already accustomed to RaceRunning and participated based on interest and motivation which may have favorably affect our results.

## Conclusions

A pre-post intervention study of 12 weeks of RaceRunning training improves cardiorespiratory endurance in individuals with CP. Moreover, RaceRunning stimulates skeletal muscle hypertrophy of the calf muscle. These results speak in favor of RaceRunning as a powerful and effective training modality in individuals with CP promoting both central and peripheral adaptations.

## Supplementary information

**Additional file 1: Figure S1.** Study participant running with the Racerunner. Photo used with permission.

**Additional file 2: Table S1.** Distribution of spasticity in the lower limb muscles assessed with the modified Ashworth scale and graded as 0, 1, +1, 2, 3, or 4, where 0 indicates no increase in muscle tone and 4 indicates marked increase in muscle tone, before and after a training period with RaceRunning (p-level 0.05).

## Data Availability

The datasets generated and analyzed during the current study are not publicly available due risk of compromising individual privacy but are available from the corresponding author on reasonable request.

## Abbreviations

6-MRT: 6-min RaceRunning test
ACSM: American College of Sports Medicine
Borg-RPE: Borg scale for rated perceived exertion
Bpm: Beats per minute
CP: Cerebral Palsy
CSA: Cross-sectional area
GMFCS: Gross Motor Function Classification System
pROM: Passive range of motion
TD: Typically developing
VO_2max_: Maximum oxygen uptake
