# Supplementary figures and images for: RaceRunning training improves stamina and promotes skeletal muscle hypertrophy in young individuals with cerebral palsy
Source: BMC Musculoskelet Disord. 2020 Mar 27;21:193. doi: 10.1186/s12891-020-03202-8 (PMC7102439; doi:10.1186/s12891-020-03202-8)

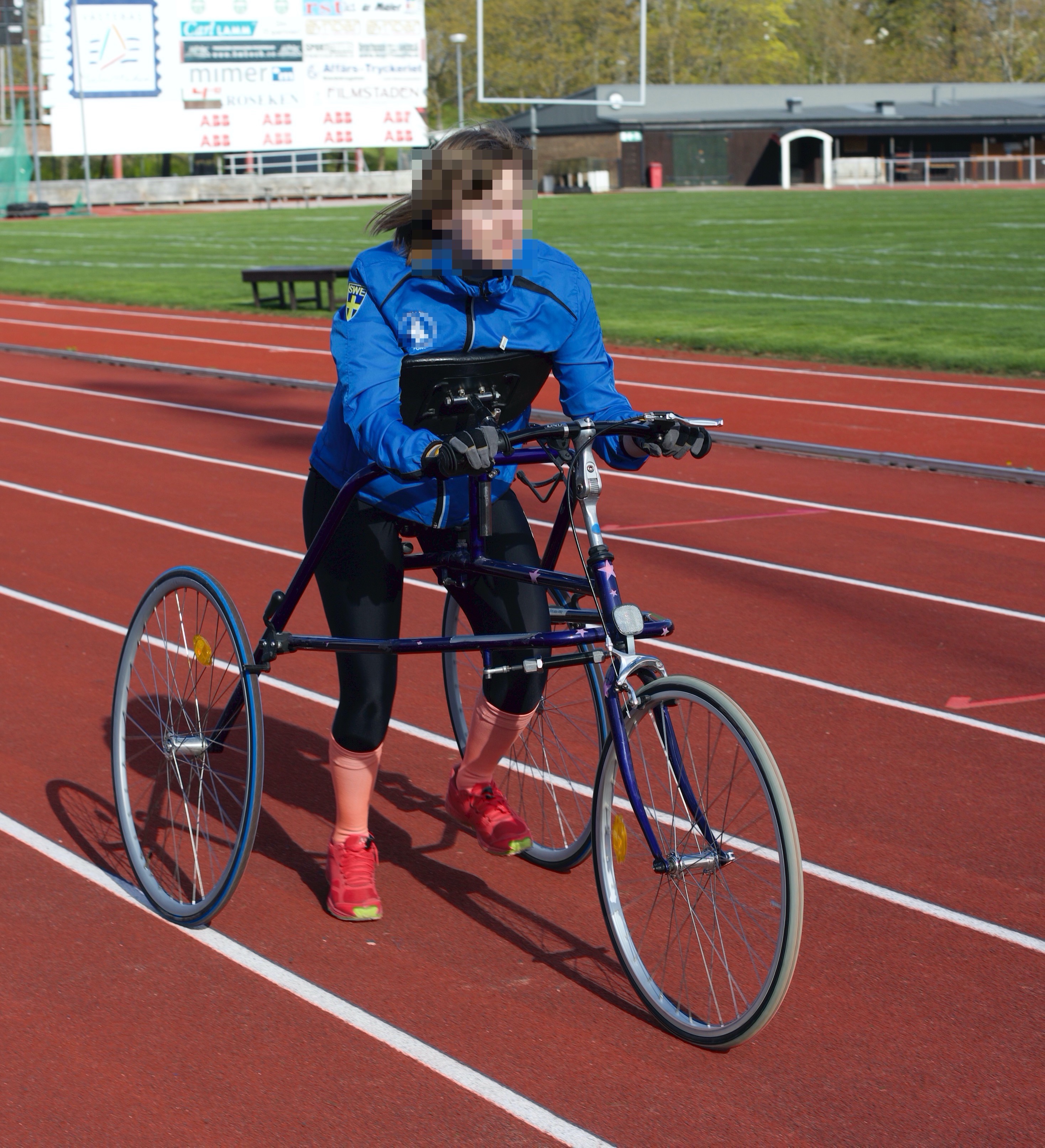

Supplement: Supplementary file 1 — Additional file 1: Figure S1. Study participant running with the Racerunner. Photo used with permission. [file 12891_2020_3202_MOESM1_ESM.zip › _RR_athelete1R2.jpg]
