# Supplementary material for: RaceRunning training improves stamina and promotes skeletal muscle hypertrophy in young individuals with cerebral palsy
Source: BMC Musculoskelet Disord. 2020 Mar 27;21:193. doi: 10.1186/s12891-020-03202-8 (PMC7102439; doi:10.1186/s12891-020-03202-8)
Supplement: Supplementary file 2 — Additional file 2: Table S1. Distribution of spasticity in the lower limb muscles assessed with the modified Ashworth scale and graded as 0, 1, +1, 2, 3, or 4, where 0 indicates no increase in muscle tone and 4 indicates marked increase in muscle tone, before and after a training period with RaceRunning (p-level 0.05). [file 12891_2020_3202_MOESM2_ESM.docx]

**Supplementary Table 1**

|  | Spasticity in more-affected leg  Number of subjects in each  Ashworth category 0/1/ +1/2/ 3/4 | | | | | Spasticity in less-affected leg  Number of subjects in each  Ashworth category 0/1/ +1/2/ 3/4 | | | | |
| --- | --- | --- | --- | --- | --- | --- | --- | --- | --- | --- |
|  | Before | n | After | n | *p-value* | Before | n | After | n | *p-value* |
| Hip flexors | 7/7/0/0/0/0 | 14 | 7/5/2/0/0/0 | 14 | 0.466 | 7/6/1/0/0/0 | 14 | 7/3/4/0/0/0 | 14 | 0.292 |
| Hip extensors | 9/0/1/3/1/0 | 14 | 10/0/0/3/1/0 | 14 | 1.000 | 9/0/0/4/1/0 | 14 | 9/1/0/3/1/0 | 14 | 1.000 |
| Knee flexors | 3/3/6/1/0/0 | 13 | 3/4/5/2/0/0 | 14 | 1.000 | 4/4/5/0/1/0 | 14 | 5/1/5/3/0/0 | 14 | 0.240 |
| Knee extensors | 10/0/0/3/1/0 | 14 | 10/0/1/2/1/0 | 14 | 1.000 | 9/0/1/1/2/0 | 13 | 10/0/1/2/1/0 | 14 | 1.000 |
| Ankle plantarflexors | 0/5/6/3/0/0 | 14 | 0/7/4/2/1/0 | 14 | 0.688 | 2/8/2/1/1/0 | 14 | 2/5/5/2/0/0 | 14 | 0.541 |

**Supplementary table 1** Distribution of spasticity in the lower limb muscles assessed with the modified Ashworth scale and graded as 0, 1, +1, 2, 3, or 4, where 0 indicates no increase in muscle tone and 4 indicates marked increase in muscle tone, before and after a training period with RaceRunning (p-level 0.05).
